# Supplementary material for: Identification of control targets in Boolean molecular network models via computational algebra
Source: BMC Syst Biol. 2016 Sep 23;10:94. doi: 10.1186/s12918-016-0332-x (PMC5035508; doi:10.1186/s12918-016-0332-x)
Supplement: Additional file 1 — Supporting material for: Identification of control targets in Boolean molecular network models via computational algebra. (PDF 214 KB) [file 12918_2016_332_MOESM1_ESM.pdf]

Supporting material for: Identification of control targets in Boolean molecular network models via computational algebra

David Murrugarra[1] Alan Veliz-Cuba[2,3] Boris Aguilar[4] Reinhard Laubenbacher[5]

[1]Department of Mathematics, University of Kentucky,
Lexington, KY 40506-0027 USA.
[2]Department of Mathematics, University of Dayton,
Dayton, OH 45469, USA.
[4]Institute for Systems Biology,
Seattle, WA 98109-5263 USA.
[5]Center for Quantitative Medicine, University of Connecticut Health Center,
Farmington, CT 06030-6033 USA.

1 A Method for Checking Reachability

In this section we describe an algebraic technique for checking the global reachability of a desired attractor using the controllers identified with the methods described in Section 3 of the paper. These methods are similar to those described in [2].

Consider $\mathbf{x}_0, \mathbf{y}_0 \in \mathbb{K}^n$ as in Section 3 of the paper, that is, $\mathbf{y}_0$ is the newly created desired attractor, and set the following ideal

$$I^{\mu}_{\mathbf{y}_0} = \langle f^{\mu}_1(x_1, \dots, x_m) - y_{01}, \dots, f^{\mu}_m(x_1, \dots, x_m) - y_{0n} \rangle \tag{1}$$

defined over the quotient ring $\mathbb{F}_2[x_1, \dots, x_m]/J$, where $J = \langle x_1^2 - x_1, \dots, x_m^2 - x_m \rangle$.

Notice that the variety $V(I^{\mu}_{\mathbf{y}_0})$ of the ideal $I^{\mu}_{\mathbf{y}_0}$ represents all predecessor states of $\mathbf{y}_0$, that is, all the states $\mathbf{x} \in \mathbb{K}^n$ such that $\mathbf{F}_\mu(\mathbf{x}) = \mathbf{y}_0$. Let $\mathbf{F}_{\mu^r}(\mathbf{x}) = \mathbf{F}_\mu(\cdots(\mathbf{F}_\mu(\mathbf{x})))$ (r times composition) and let $f^{\mu^r}_j(x_1, \dots, x_m)$ be the jth-component function of $\mathbf{F}_{\mu^r}(\mathbf{x})$. Then define

$$I^{\mu^r}_{\mathbf{y}_0} = \langle f^{\mu^r}_1(x_1, \dots, x_m) - y_{01}, \dots, f^{\mu^r}_m(x_1, \dots, x_m) - y_{0n} \rangle \tag{2}$$

Similarly, the variety $V(I^{\mu^r}_{\mathbf{y}_0})$ of the ideal $I^{\mu^r}_{\mathbf{y}_0}$ represents all states that are r-steps away from $\mathbf{y}_0$, that is, all the states $\mathbf{x} \in \mathbb{K}^n$ such that $\mathbf{F}_{\mu^r}(\mathbf{x}) = \mathbf{y}_0$.

Proposition 1.1. *Let $\boldsymbol{x}_0, \boldsymbol{y}_0 \in \mathbb{K}^n$. Then $\boldsymbol{y}_0$ is reachable from $\boldsymbol{x}_0$ if $\boldsymbol{x}_0 \in V(I^{\mu^r}_{\mathbf{y}_0})$ for some $r \geq 1$.*

Proof. It follows from the definition of $V(I^{\mu^r}_{\mathbf{y}_0})$. □

Now consider the ideals

$$J_1 = I^{\mu}_{\mathbf{y}_0} \text{ and } J_r = I^{\mu^r}_{\mathbf{y}_0} J_{r-1}, \text{ for } r = 2, \dots, n. \tag{3}$$

Notice that the variety $V(J_r)$ represents all the states that are at most r-steps ways from $\mathbf{y}_0$. It is easy to see that

$$J_1 \supset J_2 \supset \cdots \supset J_r \supset J_{r+1} \supset \cdots \tag{4}$$

Since $\mathbb{K}^n$ is finite, the descending chain in Equation 4 stops at some index $r = N$. That is,

$$J_N = J_{N+1} = J_{N+2} = \cdots \tag{5}$$

The following proposition uses equations 4-5 to establish a test for global reachability of the new attractor $\mathbf{y}_0$.

Proposition 1.2. *Let $\boldsymbol{y}_0 \in \mathbb{K}^n$. Then $\boldsymbol{y}_0$ is globally reachable if the chain in Equation 5 stops at the zero-ideal. That is,*

$$J_N = J_{N+1} = \{0\}. \tag{6}$$

Proof. It follows from the fact that $V(\{0\}) = \mathbb{K}^n$. □

2 P53-mdm2 Network Polynomials

Consider the network at Figure 2 of the main text for the signaling pathway of *p53* that was published in [1]. This is a discrete dynamical system $\mathbf{F} = (f_1, \ldots, f_{16}) : \mathbb{F}_2^{16} \to \mathbb{F}_2^{16}$ with 16 nodes and binary states $\mathbb{F}_2 = \{0, 1\}$. Let us represent the nodes by

$x_1 = ATM$,	$x_2 = p53$,
$x_3 = Mdm2$,	$x_4 = MdmX$,
$x_5 = Wip1$,	$x_6 = cyclinG$,
$x_7 = PTEN$,	$x_8 =$ p21,
$x_9 = AKT$,	$x_{10} = cyclinE$,
$x_{11} = Rb$,	$x_{12} = E2F1$,
$x_{13} = p14ARf$,	$x_{14} = Bcl2$,
$x_{15} = Bax$,	$x_{16} = caspase$.

The polynomial functions for this network are given below. For the cancer cell model where *PTEN* and *p14ARf* are inactive (fixed to zero) and *cyclinG* is always active (fixed to 1) make the value of these variables equal to the corresponding constant.

f1 = 1+x5+x1*x5+x12*x5+x1*x12*x5+x6+x1*x6+x12*x6+x1*x12*x6+x5*x6,
f2 = 1+x3+x1*x2*x3+x4+x1*x4+x3*x4+x1*x3*x4+x1*x2*x3*x4,
f3 = 1+x1+x10+x1*x10+x11+x1*x11+x10*x11+x1*x10*x11+x13+x1*x13+x10*x13+
x1*x10*x13+x11*x13+x1*x11*x13+x10*x11*x13+x1*x10*x11*x13+x1*x2*x3+x10*x2*x3+
x11*x2*x3+x1*x10*x11*x2*x3+x1*x13*x2*x3+x10*x13*x2*x3+x11*x13*x2*x3+
x1*x10*x11*x13*x2*x3+x1*x4+x10*x4+x11*x4+x1*x10*x11*x4+x1*x13*x4+x10*x13*x4+
x11*x13*x4+x1*x10*x11*x13*x4+x13*x2*x4+x1*x13*x2*x4+x10*x13*x2*x4+
x1*x10*x13*x2*x4+x11*x13*x2*x4+x1*x11*x13*x2*x4+x10*x11*x13*x2*x4+
x1*x10*x11*x13*x2*x4+x13*x3*x4+x1*x13*x3*x4+x10*x13*x3*x4+x1*x10*x13*x3*x4+
x11*x13*x3*x4+x1*x11*x13*x3*x4+x10*x11*x13*x3*x4+x1*x10*x11*x13*x3*x4+
x1*x2*x3*x4+x10*x2*x3*x4+x1*x10*x2*x3*x4+x11*x2*x3*x4+x1*x11*x2*x3*x4+
x10*x11*x2*x3*x4+x13*x2*x3*x4+x1*x10*x11*x13*x2*x3*x4+x1*x5+x10*x5+
x11*x5+x1*x10*x11*x5+x1*x13*x5+x10*x13*x5+x11*x13*x5+x1*x10*x11*x13*x5+
x13*x2*x5+x1*x13*x2*x5+x10*x13*x2*x5+x1*x10*x13*x2*x5+x11*x13*x2*x5+
x1*x11*x13*x2*x5+x10*x11*x13*x2*x5+x1*x10*x11*x13*x2*x5+x13*x3*x5+x1*x13*x3*x5+
x10*x13*x3*x5+x1*x10*x13*x3*x5+x11*x13*x3*x5+x1*x11*x13*x3*x5+x10*x11*x13*x3*x5+
x1*x10*x11*x13*x3*x5+x1*x2*x3*x5+x10*x2*x3*x5+x1*x10*x2*x3*x5+x11*x2*x3*x5+
x1*x11*x2*x3*x5+x10*x11*x2*x3*x5+x13*x2*x3*x5+x1*x10*x11*x13*x2*x3*x5+x1*x4*x5+
x10*x4*x5+x1*x10*x4*x5+x11*x4*x5+x1*x11*x4*x5+x10*x11*x4*x5+x13*x4*x5+
x1*x10*x11*x13*x4*x5+x1*x13*x2*x4*x5+x10*x13*x2*x4*x5+x11*x13*x2*x4*x5+
x1*x10*x11*x13*x2*x4*x5+x1*x13*x3*x4*x5+x10*x13*x3*x4*x5+
x11*x13*x3*x4*x5+x1*x10*x11*x13*x3*x4*x5+x1*x2*x3*x4*x5+x10*x2*x3*x4*x5+
x11*x2*x3*x4*x5+x1*x10*x11*x13*x2*x3*x4*x5+x1*x2*x6+x10*x2*x6+x11*x2*x6+
x1*x10*x11*x2*x6+x1*x13*x2*x6+x10*x13*x2*x6+x11*x13*x2*x6+x1*x10*x11*x13*x2*x6+
x1*x3*x6+x10*x3*x6+x11*x3*x6+x1*x10*x11*x3*x6+x1*x13*x3*x6+x10*x13*x3*x6+
x11*x13*x3*x6+x1*x10*x11*x13*x3*x6+x13*x2*x3*x6+x1*x13*x2*x3*x6+
x10*x13*x2*x3*x6+x1*x10*x13*x2*x3*x6+x11*x13*x2*x3*x6+x1*x11*x13*x2*x3*x6+

x10*x11*x13*x2*x3*x6+x1*x10*x11*x13*x2*x3*x6+x13*x4*x6+x1*x13*x4*x6+
x10*x13*x4*x6+x1*x10*x13*x4*x6+x11*x13*x4*x6+
x1*x11*x13*x4*x6+x10*x11*x13*x4*x6+x1*x10*x11*x13*x4*x6+x1*x2*x4*x6+
x10*x2*x4*x6+x1*x10*x2*x4*x6+x11*x2*x4*x6+x1*x11*x2*x4*x6+x10*x11*x2*x4*x6+
x13*x2*x4*x6+x1*x10*x11*x13*x2*x4*x6+x1*x3*x4*x6+
x10*x3*x4*x6+x1*x10*x3*x4*x6+x11*x3*x4*x6+x1*x11*x3*x4*x6+x10*x11*x3*x4*x6+
x13*x3*x4*x6+x1*x10*x11*x13*x3*x4*x6+x1*x13*x2*x3*x4*x6+
x10*x13*x2*x3*x4*x6+x11*x13*x2*x3*x4*x6+x1*x10*x11*x13*x2*x3*x4*x6+x13*x5*x6+
x1*x13*x5*x6+x10*x13*x5*x6+x1*x10*x13*x5*x6+x11*x13*x5*x6+x1*x11*x13*x5*x6+
x10*x11*x13*x5*x6+x1*x10*x11*x13*x5*x6+x1*x2*x5*x6+x10*x2*x5*x6+x1*x10*x2*x5*x6+
x11*x2*x5*x6+x1*x11*x2*x5*x6+x10*x11*x2*x5*x6+x13*x2*x5*x6+
x1*x10*x11*x13*x2*x5*x6+x1*x3*x5*x6+x10*x3*x5*x6+x1*x10*x3*x5*x6+
x11*x3*x5*x6+x1*x11*x3*x5*x6+x10*x11*x3*x5*x6+x13*x3*x5*x6+x1*x10*x11*x13*x3*x5*x6+
x1*x13*x2*x3*x5*x6+x10*x13*x2*x3*x5*x6+x11*x13*x2*x3*x5*x6+x1*x10*x11*x13*x2*x3*x5*x6+
x1*x13*x4*x5*x6+x10*x13*x4*x5*x6+x11*x13*x4*x5*x6+x1*x10*x11*x13*x4*x5*x6+
x1*x2*x4*x5*x6+x10*x2*x4*x5*x6+x11*x2*x4*x5*x6+
x1*x10*x11*x13*x2*x4*x5*x6+x1*x3*x4*x5*x6+x10*x3*x4*x5*x6+x11*x3*x4*x5*x6+
x1*x10*x11*x13*x3*x4*x5*x6+x13*x2*x3*x4*x5*x6+x1*x10*x11*x13*x2*x3*x4*x5*x6+
x1*x2*x9+x10*x2*x9+x11*x2*x9+x1*x10*x11*x2*x9+x1*x13*x2*x9+
x10*x13*x2*x9+x11*x13*x2*x9+x1*x10*x11*x13*x2*x9+x1*x3*x9+x10*x3*x9+x11*x3*x9+
x1*x10*x11*x3*x9+x1*x13*x3*x9+x10*x13*x3*x9+x11*x13*x3*x9+
x1*x10*x11*x13*x3*x9+x13*x2*x3*x9+x1*x13*x2*x3*x9+x10*x13*x2*x3*x9+
x1*x10*x13*x2*x3*x9+x11*x13*x2*x3*x9+
x1*x11*x13*x2*x3*x9+x10*x11*x13*x2*x3*x9+
x1*x10*x11*x13*x2*x3*x9+x13*x4*x9+x1*x13*x4*x9+x10*x13*x4*x9+
x1*x10*x13*x4*x9+x11*x13*x4*x9+
x1*x11*x13*x4*x9+x10*x11*x13*x4*x9+x1*x10*x11*x13*x4*x9+x1*x2*x4*x9+x10*x2*x4*x9+
x1*x10*x2*x4*x9+x11*x2*x4*x9+x1*x11*x2*x4*x9+x10*x11*x2*x4*x9+x13*x2*x4*x9+
x1*x10*x11*x13*x2*x4*x9+x1*x3*x4*x9+x10*x3*x4*x9+x1*x10*x3*x4*x9+x11*x3*x4*x9+
x1*x11*x3*x4*x9+x10*x11*x3*x4*x9+x13*x3*x4*x9+x1*x10*x11*x13*x3*x4*x9+
x1*x13*x2*x3*x4*x9+x10*x13*x2*x3*x4*x9+x11*x13*x2*x3*x4*x9+
x1*x10*x11*x13*x2*x3*x4*x9+x13*x5*x9+x1*x13*x5*x9+x10*x13*x5*x9+x1*x10*x13*x5*x9+
x11*x13*x5*x9+x1*x11*x13*x5*x9+x10*x11*x13*x5*x9+x1*x10*x11*x13*x5*x9+x1*x2*x5*x9+
x10*x2*x5*x9+x1*x10*x2*x5*x9+x11*x2*x5*x9+x1*x11*x2*x5*x9+x10*x11*x2*x5*x9+
x13*x2*x5*x9+x1*x10*x11*x13*x2*x5*x9+x1*x3*x5*x9+x10*x3*x5*x9+x1*x10*x3*x5*x9+
x11*x3*x5*x9+x1*x11*x3*x5*x9+x10*x11*x3*x5*x9+x13*x3*x5*x9+
x1*x10*x11*x13*x3*x5*x9+x1*x13*x2*x3*x5*x9+x10*x13*x2*x3*x5*x9+
x11*x13*x2*x3*x5*x9+x1*x10*x11*x13*x2*x3*x5*x9+x1*x13*x4*x5*x9+x10*x13*x4*x5*x9+
x11*x13*x4*x5*x9+x1*x10*x11*x13*x4*x5*x9+x1*x2*x4*x5*x9+x10*x2*x4*x5*x9+
x11*x2*x4*x5*x9+x1*x10*x11*x13*x2*x4*x5*x9+x1*x3*x4*x5*x9+x10*x3*x4*x5*x9+
x11*x3*x4*x5*x9+x1*x10*x11*x13*x3*x4*x5*x9+x13*x2*x3*x4*x5*x9+
x1*x10*x11*x13*x2*x3*x4*x5*x9+x1*x6*x9+x10*x6*x9+x11*x6*x9+x1*x10*x11*x6*x9+
x1*x13*x6*x9+x10*x13*x6*x9+x11*x13*x6*x9+x1*x10*x11*x13*x6*x9+x13*x2*x6*x9+
x1*x13*x2*x6*x9+x10*x13*x2*x6*x9+x1*x10*x13*x2*x6*x9+x11*x13*x2*x6*x9+
x1*x11*x13*x2*x6*x9+x10*x11*x13*x2*x6*x9+x1*x10*x11*x13*x2*x6*x9+x13*x3*x6*x9+
x1*x13*x3*x6*x9+x10*x13*x3*x6*x9+x1*x10*x13*x3*x6*x9+x11*x13*x3*x6*x9+
x1*x11*x13*x3*x6*x9+x10*x11*x13*x3*x6*x9+x1*x10*x11*x13*x3*x6*x9+
x1*x2*x3*x6*x9+x10*x2*x3*x6*x9+x1*x10*x2*x3*x6*x9+x11*x2*x3*x6*x9+
x1*x11*x2*x3*x6*x9+x10*x11*x2*x3*x6*x9+x13*x2*x3*x6*x9+x1*x10*x11*x13*x2*x3*x6*x9+
x1*x4*x6*x9+x10*x4*x6*x9+x1*x10*x4*x6*x9+x11*x4*x6*x9+x1*x11*x4*x6*x9+

x10*x11*x4*x6*x9+x13*x4*x6*x9+x1*x10*x11*x13*x4*x6*x9+x1*x13*x2*x4*x6*x9+
x10*x13*x2*x4*x6*x9+x11*x13*x2*x4*x6*x9+x1*x10*x11*x13*x2*x4*x6*x9+
x1*x13*x3*x4*x6*x9+x10*x13*x3*x4*x6*x9+x11*x13*x3*x4*x6*x9+
x1*x10*x11*x13*x3*x4*x6*x9+x1*x2*x3*x4*x6*x9+x10*x2*x3*x4*x6*x9+
x11*x2*x3*x4*x6*x9+x1*x10*x11*x13*x2*x3*x4*x6*x9+
x1*x5*x6*x9+x10*x5*x6*x9+x1*x10*x5*x6*x9+x11*x5*x6*x9+x1*x11*x5*x6*x9+
x10*x11*x5*x6*x9+x13*x5*x6*x9+x1*x10*x11*x13*x5*x6*x9+x1*x13*x2*x5*x6*x9+
x10*x13*x2*x5*x6*x9+x11*x13*x2*x5*x6*x9+x1*x10*x11*x13*x2*x5*x6*x9+
x1*x13*x3*x5*x6*x9+x10*x13*x3*x5*x6*x9+x11*x13*x3*x5*x6*x9+
x1*x10*x11*x13*x3*x5*x6*x9+x1*x2*x3*x5*x6*x9+x10*x2*x3*x5*x6*x9+
x11*x2*x3*x5*x6*x9+x1*x10*x11*x13*x2*x3*x5*x6*x9+x1*x4*x5*x6*x9+
x10*x4*x5*x6*x9+x11*x4*x5*x6*x9+x1*x10*x11*x13*x4*x5*x6*x9+
x13*x2*x4*x5*x6*x9+x1*x10*x11*x13*x2*x4*x5*x6*x9+x13*x3*x4*x5*x6*x9+
x1*x10*x11*x13*x3*x4*x5*x6*x9+x1*x2*x3*x4*x5*x6*x9+x10*x2*x3*x4*x5*x6*x9+
x1*x10*x2*x3*x4*x5*x6*x9+x11*x2*x3*x4*x5*x6*x9+x1*x11*x2*x3*x4*x5*x6*x9+
x10*x11*x2*x3*x4*x5*x6*x9+x1*x10*x11*x2*x3*x4*x5*x6*x9+x13*x2*x3*x4*x5*x6*x9+
x1*x13*x2*x3*x4*x5*x6*x9+x10*x13*x2*x3*x4*x5*x6*x9+
x1*x10*x13*x2*x3*x4*x5*x6*x9+x11*x13*x2*x3*x4*x5*x6*x9+
x1*x11*x13*x2*x3*x4*x5*x6*x9+x10*x11*x13*x2*x3*x4*x5*x6*x9,
f4 = 1+x1+x13+x1*x13+x3+x1*x3+x13*x3+x1*x13*x3+x1*x4*x5+x1*x13*x4*x5+
x1*x3*x4*x5+x1*x13*x3*x4*x5+x1*x9+x1*x13*x9+x1*x3*x9+x1*x13*x3*x9+
x13*x4*x9+x1*x13*x4*x9+x13*x3*x4*x9+x1*x13*x3*x4*x9+x13*x5*x9+
x1*x13*x5*x9+x13*x3*x5*x9+x1*x13*x3*x5*x9+x1*x4*x5*x9+
x13*x4*x5*x9+x3*x4*x5*x9+x1*x13*x3*x4*x5*x9,
f5 = x2,
f6 = x2,
f7 = x2,
f8 = x2 + x2*x3*x9 + x2*x3*x8*x9,
f9 = 1+x7,
f10 = 1+x8,
f11 = x1+x11+x1*x11+x10*x11+x1*x10*x11+x1*x16+x11*x16+x1*x11*x16+x10*x11*x16+
x1*x10*x11*x16+x1*x10*x3+x11*x3+x1*x11*x3+x10*x11*x3+x1*x10*x16*x3+x11*x16*x3+
x1*x11*x16*x3+x10*x11*x16*x3,
f12 = 1+x11+x1*x11+x11*x12+x1*x11*x12+x13+x11*x13+x1*x11*x13+x11*x12*x13+
x1*x11*x12*x13+x11*x3+x1*x11*x3+x11*x12*x3+x1*x11*x12*x3+x11*x13*x3+
x1*x11*x13*x3+x1*x12*x13*x3+x11*x12*x13*x3,
f13 = x12+x13+x12*x13+x12*x2+x13*x2+x12*x13*x2+x12*x5+x13*x5+
x12*x2*x5+x13*x2*x5,
f14 = x14+x14*x16+x14*x2+x14*x16*x2+x9+x14*x9+x14*x16*x9+x2*x9,
f15 = x2 + x14*x2 + x14*x15*x2,
f16 = x15+x12*x16+x12*x14*x16+x12*x15*x16+x12*x14*x15*x16+x14*x15*x8+
x12*x14*x15*x8+x12*x16*x8+x12*x14*x16*x8+x12*x15*x16*x8+
x14*x15*x16*x8+x14*x15*x9+x12*x14*x15*x9+x12*x16*x9+
x12*x14*x16*x9+x12*x15*x16*x9+x14*x15*x16*x9+
x15*x8*x9+x12*x15*x8*x9+x12*x14*x15*x8*x9+x12*x16*x8*x9+
x12*x14*x16*x8*x9+x15*x16*x8*x9+x14*x15*x16*x8*x9+x12*x14*x15*x16*x8*x9.

3 P53-mdm2 network control combinations

Here we list the top 10 controllers for the cancer cell model where $PTEN$ and $p14ARf$ are inactive (fixed to zero) and $cyclinG$ is always active (fixed to 1), see Table 5 of [1].

The 10 control sets that give the largest basin for $\mathbf{y}_0$ are given in Table 1 and the 10 control sets that give the smallest basin for $\mathbf{y}_0$ are in Table 2. For each solution, the edges that are crossed-out (in red) are edges that become nonessential after the other controllers in the set have been applied.

Sets	Controllers	Basin %
1	$p53 \to Wip1$, $Mdm2 \to p21$, $Mdm2 \to p53$, $p21 \to Caspase$.	60.815
2	$p53 \to Wip1$, $Mdm2 \to p21$, ~~$p53 \to p53$~~, $Mdm2 \to p53$, $p21 \to Caspase$.	60.815
3	$p53 \to Wip1$, ~~$p21 \to p21$~~, $Mdm2 \to p21$, $Mdm2 \to p53$, $p21 \to Caspase$.	60.815
4	$p53 \to Wip1$, ~~$p21 \to p21$~~, $Mdm2 \to p21$, ~~$p53 \to p53$~~, $Mdm2 \to p53$, $p21 \to Caspase$.	60.815
5	$p53 \to Wip1$, $ATM \to Rb$, $Mdm2 \to p21$, $Mdm2 \to p53$, $p21 \to Caspase$.	60.815
6	$p53 \to Wip1$, $ATM \to Rb$, $Mdm2 \to p21$, ~~$p53 \to p53$~~, $Mdm2 \to p53$, $p21 \to Caspase$, $p21 \to Caspase$.	60.815
7	$p53 \to Wip1$, $ATM \to Rb$, ~~$p21 \to p21$~~, $Mdm2 \to p21$, $Mdm2 \to p53$, $p21 \to Caspase$.	60.815
8	$p53 \to Wip1$, $ATM \to Rb$, ~~$p21 \to p21$~~, $Mdm2 \to p21$, ~~$p53 \to p53$~~, $Mdm2 \to p53$, $p21 \to Caspase$.	60.815
9	$p53 \to Wip1$, $Caspase \to Rb$, $ATM \to Rb$, $Mdm2 \to p21$, $Mdm2 \to p53$, $p21 \to Caspase$.	60.815
10	$p53 \to Wip1$, $Caspase \to Rb$, $ATM \to Rb$, $Mdm2 \to p21$, ~~$p53 \to p53$~~, $Mdm2 \to p53$, $p21 \to Caspase$.	60.815

Table 1: Control sets that give the largest basin for $\mathbf{y}_0$.

Sets	Controllers	Basin %
936	$p53 \to Wip1$, $Mdm2 \to p21$, $ATM \to ATM$, $E2F1 \to Caspase$.	0.42725
937	$p53 \to Wip1$, ~~$p21 \to p21$~~, $Mdm2 \to p21$, $ATM \to ATM$, $E2F1 \to Caspase$.	0.42725
938	$p53 \to Wip1$, $ATM \to Rb$, $Mdm2 \to p53$, ~~$ATM \to p53$~~, $E2F1 \to ATM$, $E2F1 \to Caspase$.	0.41504
939	$p53 \to Wip1$, $ATM \to Rb$, ~~$p53 \to p53$~~, $Mdm2 \to p53$, ~~$ATM \to p53$~~, $E2F1 \to ATM$, $E2F1 \to Caspase$.	0.41504
940	$p53 \to Wip1$, $Caspase \to Rb$, $ATM \to Rb$, $ATM \to ATM$, $E2F1 \to Caspase$.	0.41504
941	$p53 \to Wip1$, $Caspase \to Rb$, $ATM \to Rb$, $Mdm2 \to p53$, ~~$ATM \to p53$~~, $E2F1 \to ATM$, $E2F1 \to Caspase$.	0.41504
942	$p53 \to Wip1$, $Caspase \to Rb$, $ATM \to Rb$, ~~$p53 \to p53$~~, $Mdm2 \to p53$, ~~$ATM \to p53$~~, $E2F1 \to ATM$, $E2F1 \to Caspase$.	0.41504
943	$p53 \to Wip1$, $ATM \to ATM$, $E2F1 \to Caspase$,	0.3418
944	$p53 \to Wip1$, $Mdm2 \to p53$, ~~$ATM \to p53$~~, $E2F1 \to ATM$, $E2F1 \to Caspase$,	0.31738
945	$p53 \to Wip1$, ~~$p53 \to p53$~~, $Mdm2 \to p53$, ~~$ATM \to p53$~~, $E2F1 \to ATM$, $E2F1 \to Caspase$.	0.31738

Table 2: Control sets that give the smallest basin for $\mathbf{y}_0$.

4 T-LGL network

Here we show details of the computation of the control targets shown in Eq. 13 (main text) and Table 2 (main text).

First, we consider the polynomials $g_i := (u_i^- + u_i^+ + 1)f_i(x) + u_i^+ - x_i$. We are interested in the solutions of the system of 18 equations $g_1 = 0, \ldots, g_{16} = 0, x_{16} = 0, x_{10} = 0$. Since the combination of node deletion and constant expression of the same node is not biologically relevant, we require that at least one of the control parameters u_i^+, u_i^- is zero; that is, we require $u_i^- u_i^+ = 0$. The algebraic representation of the solutions is then encoded as the ideal of polynomials $I = \langle g_1, g_2, \ldots, g_{16}, x_{16}, x_{10}, u_1^- u_1^+, u_2^- u_2^+, \ldots, u_{16}^- u_{16}^+ \rangle$.

Using the algebra software Macaulay2, we find that the Gröbner basis in lexicographical order of this ideal is

$G = \{u_{10}^+,\ u_{16}^+,\ (u_{10}^- + 1)u_9^+,\ (u_{10}^- + 1)(u_9^- + 1)u_8^+,\ (u_{10}^- + 1)(u_{13}^+ + 1)u_{12}^+,\ u_{15}^-(u_{10}^- + 1)(u_9^- + 1)(u_8^- + 1)(u_6^- + 1)(u_7^+ + 1),\ u_{11}^-(u_{10}^- + 1)(u_9^- + 1)u_7^-(u_6^- + 1),\ u_{14}^-(u_{12}^- + 1)(u_{10}^- + 1)(u_{13}^+ + 1),\ u_{13}^-(u_{10}^- + 1)u_{12}^+,\ u_{15}^- u_{11}^-(u_{10}^- + 1)(u_9^- + 1)(u_6^- + 1)(u_7^+ + 1),\ u_{15}^-(u_{10}^- + 1)(u_9^- + 1)(u_8^- + 1)u_7^-(u_6^- + 1),\ u_{15}^-(u_{10}^- + 1)(u_9^- + 1)(u_8^- + 1)u_6^+,\ u_{14}^- u_{13}^-(u_{12}^- + 1)(u_{10}^- + 1),\ u_{11}^-(u_{10}^- + 1)(u_9^- + 1)u_6^+, \ldots\ \}$

The polynomials not shown in the list above consist of polynomials that contain variables x_i's, or polynomials of the form $u_i^+ u_i^-$, which give no new information. We make two important remarks. First, the list of polynomials in G encode the same solutions as the ideal I. Thus, if we can choose parameters such that the system of equations defined by G has no solution, then the original system will have no solutions. Second, the polynomials shown above depend only on the control parameters. Thus, for appropriate choices of u_i^+ and u_i^-, we can make one of such polynomials equal to 1 (e.g. for $u_{10}^+ = 1$), which guarantees that the original system has no solutions regardless of the value of the variables x.

Then, the control policies that we obtain are the following:

1. $u_{16}^+ = 1$, no restriction on other controls
2. $u_{10}^+ = 1$, no restriction on other controls
3. $u_{10}^- = 0, u_9^+ = 1$, no restriction on other controls
4. $u_{10}^- = 0, u_9^- = 0, u_8^+ = 1$, no restriction on other controls
5. $u_{10}^- = 0, u_{13}^+ = 0, u_{12}^+ = 1$, no restriction on other controls
6. $u_{15}^- = 1, u_{10}^- = 0, u_9^- = 0, u_8^- = 0, u_6^- = 0, u_7^+ = 0$, no restriction on other controls
7. $u_{14}^- = 1, u_{12}^- = 0, u_{10}^- = 0, u_{13}^+ = 0$, no restriction on other controls
8. $u_{11}^- = 1, u_{10}^- = 0, u_9^- = 0, u_6^+ = 1$, no restriction on other controls
9. $u_{11}^- = 1, u_{10}^- = 0, u_9^- = 0, u_7^- = 1, u_6^- = 0$, no restriction on other controls
10. $u_{13}^- = 1, u_{10}^- = 0, u_{12}^+ = 1$, no restriction on other controls
11. $u_{15}^- = 1, u_{11}^- = 1, u_{10}^- = 0, u_9^- = 0, u_6^- = 0, u_7^+ = 0$, no restriction on other controls
12. $u_{15}^- = 1, u_{10}^- = 0, u_9^- = 0, u_8^- = 0, u_7^- = 1, u_6^- = 0$, no restriction on other controls
13. $u_{15}^- = 1, u_{10}^- = 0, u_9^- = 0, u_8^- = 0, u_6^+ = 1$, no restriction on other controls
14. $u_{14}^- = 1, u_{13}^- = 1, u_{12}^- = 0, u_{10}^- = 0$, no restriction on other controls

Note that some control policies are better than others. For example, control policy 5 needs only one active control ($u_{12}^+ = 1$, all the others can be zero), but control policy 10 requires 2 active controls ($u_{12}^+ = 1$ and $u_{13}^- = 1$). By inspection, we see that the best control policies are the following:

1. $u_{16}^+ = 1$, all other controls equal to zero

2. $u_{10}^{+} = 1$, all other controls equal to zero
3. $u_{9}^{+} = 1$, all other controls equal to zero
4. $u_{8}^{+} = 1$, all other controls equal to zero
5. $u_{12}^{+} = 1$, all other controls equal to zero
6. $u_{15}^{-} = 1$, all other controls equal to zero
7. $u_{14}^{-} = 1$, all other controls equal to zero
8. $u_{11}^{-} = 1, u_{6}^{+} = 1$, all other controls equal to zero
9. $u_{11}^{-} = 1, u_{7}^{-} = 1$, all other controls equal to zero

These are the controls reported in Table 2 (main text). Note that control $u_{16}^{+} = 1$ corresponds to the constant expression of the conceptual node x_{16} =Apoptosis, so it is not explicitly reported.

References

[1] M. Choi, J. Shi, S. H. Jung, X. Chen, and K.-H. Cho, *Attractor landscape analysis reveals feedback loops in the p53 network that control the cellular response to dna damage*, Sci. Signal., 5 (2012), p. ra83.

[2] R. Li, M. Yang, and T. Chu, *Controllability and observability of boolean networks arising from biology*, Chaos, 25 (2015), p. 023104.
